# Ultrasonographic aspects of sural nerve anatomy

**DOI:** 10.1590/0100-3984.2024.0120

**Published:** 2025-07-10

**Authors:** Márcio Luís Duarte, Ocacir de Souza Reis Soares, Jean-Louis Brasseur

**Affiliations:** 1 Universidade de Ribeirão Preto (Unaerp), Campus Guarujá, Guarujá, SP, Brazil; 2 Diagnósticos da América S.A. (Dasa), Barueri, SP, Brazil; 2 Clínica Radiológica Ocacir Soares, Presidente Prudente, SP, Brazil; 4 Hôpital de la Pitié-Salpêtrière, Paris, France

**Keywords:** Diagnosis, Sural nerve, Ultrasonography, Anatomy., Diagnóstico, Nervo sural, Ultrassonografia, Anatomia

## Abstract

The anatomy of the sural nerve is highly variable, and the nerve can present injuries of various etiologies, including iatrogenic injury during surgery. Precise knowledge of the course and morphology of the sural nerve is valuable, and the ability to assess the nerve properly before surgery increases the postoperative success rate, as well as facilitating the execution of nerve conduction studies and biopsies. The purpose of this article is to describe and illustrate the anatomy of the sural nerve, as seen on ultrasonography, which is a practical and economical imaging method.

## Abstract (pt)

A anatomia do nervo sural é altamente variável e o nervo pode apresentar lesões de várias etiologias, incluindo lesão iatrogênica durante cirurgia. O conhecimento preciso do curso e da morfologia do nervo sural é valioso, e a capacidade de avaliar o nervo adequadamente antes da cirurgia aumenta a taxa de sucesso pós-operatório, bem como facilita a execução de estudos de condução nervosa e biópsias. O objetivo deste artigo é descrever e ilustrar a anatomia do nervo sural, conforme demonstrada na ultrassonografia, que é um método de imagem prático e econômico.

## INTRODUCTION

The sural nerve, also referred to as the short saphenous nerve, is a superficial sensory nerve of the leg that courses along the posterior aspect of the calf. It typically originates from the distal half of the leg and traverses superficially between the two heads of the gastrocnemius muscle^(1)^. The sural nerve branches to innervate the skin on the posterolateral distal third of the leg, ultimately emerging as the lateral dorsal cutaneous nerve that innervates the lateral aspect of the foot, including the lateral aspect of the fifth toe. The sural nerve also innervates the lateral aspect of the heel through the lateral calcaneal branch^(1)^.

The sural nerve is widely used for general diagnostic purposes, such as in nerve conduction studies and biopsies, as well as for therapeutic purposes, such as in nerve grafting procedures^(2)^. However, because of its superficial location, the sural nerve is subject to iatrogenic injury, even during minimally invasive surgical procedures, and damage to this nerve may have effects on patient quality of life, provoking symptoms ranging from sensory disturbances to severe pain, often accompanied by neuromas or even complete sensory loss^(3)^.

Technological advances have made ultrasonography a preferred imaging method for evaluating peripheral nerves^(4)^. Ultrasound imaging of the sural nerve and reference sites has been used for nerve block injection under ultrasound guidance^(4)^. Ultrasonography can also facilitate the planning of surgical procedures^(5)^. The sural nerve is a preferred biopsy target because it is an easily accessible pure sensory nerve that is often affected in cases of vasculitic peripheral neuropathy. The risk of sensory deficit from sural nerve biopsy is relatively low; because of its dorsal innervation, biopsy of this nerve is unlikely to lead to pressure ulcers^(6)^. In addition, ultrasound mapping is relatively inexpensive, does not involve radiation, and can be performed at the bedside or in the operating room, with minimal logistical requirements^(6)^.

Ultrasound analysis of the two components of the sural nerve-the medial sural cutaneous nerve (MSCN) and the lateral sural cutaneous nerve (LSCN)-may have other applications^(1^,^5^,^6)^: diagnostic (for biopsy and nerve conduction studies); and therapeutic (for nerve grafting, in which the sural nerve can be sacrificed for the reconstruction of a functionally more important nerve, in cases of paralysis of the facial nerve or postoperative erectile dysfunction after radical prostatectomy). The LSCN, a branch of the common peroneal nerve that does not exist in every individual, can also be used to create a sensate free flap; a detailed knowledge of the anatomy of the sural nerve and of the nerves that contribute to it is important for the execution of such procedures^(5)^.

## ANATOMY AND ULTRASONOGRAPHIC CHARACTERISTICS OF THE SURAL NERVE

**Figure 1:**
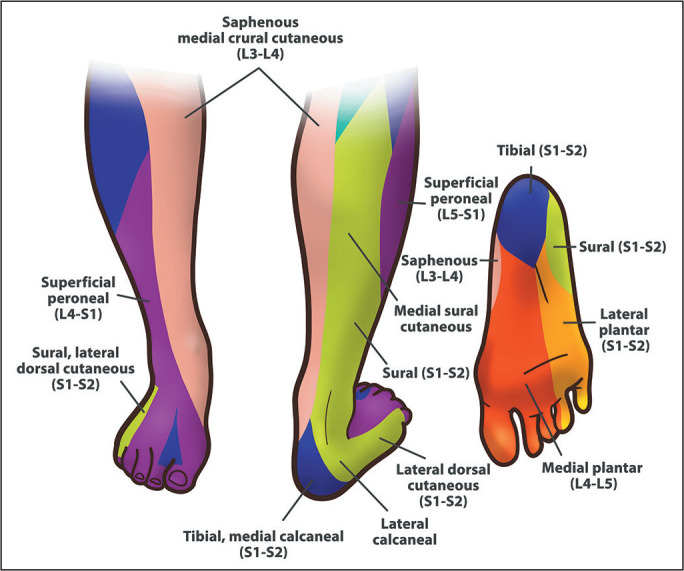
Sensory distribution of the distal sural nerve.

**Figure 2:**
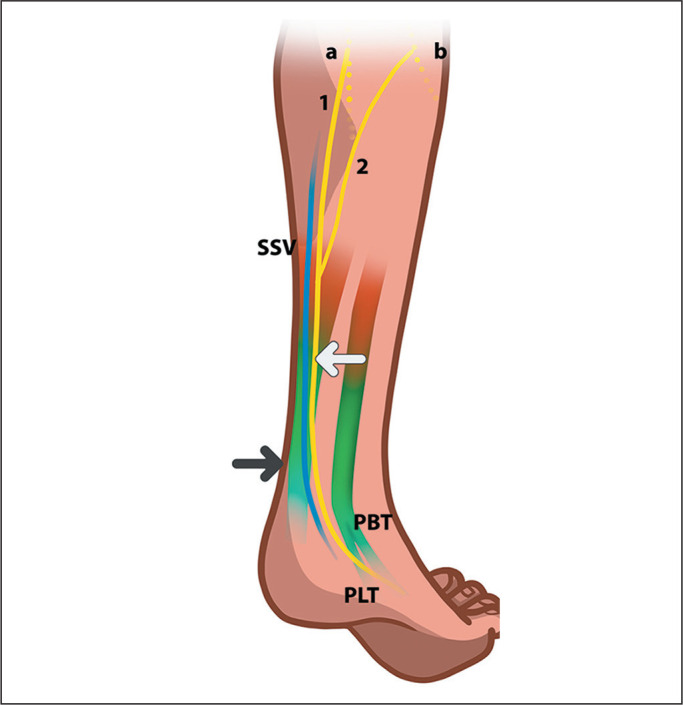
Anatomical distribution of the distal sural nerve. In the proximal portion of the calf, the tibial (a) and common peroneal (b) nerves give rise to the roots of the sural nerve (white arrow), including the medial and lateral sural cutaneous branches (1 and 2, respectively). Those two roots join at the middle or distal third of the calf. The sural nerve runs distally near the small saphenous vein (SSV) in a close relationship with the calcaneal tendon (black arrow). In the distal portion of the leg, the nerve is located between the calcaneal tendon and the peroneus longus tendon (PLT) and peroneus brevis tendon (PBT).

As illustrated in Figure 1, the sural nerve is a sensory nerve that innervates the skin of the posterolateral portion of the distal third of the leg, the lateral portion of the calcaneus, and the side of the foot^(4)^. It is generally thought to originate distal to the popliteal fossa and descend between the medial and lateral heads of the gastrocnemius muscle (Figure 2), often being accompanied by the small saphenous vein and formed by the union of the MSCN (a branch of the tibial nerve) and the LSCN^(3^,^4)^.

**Figure 3:**
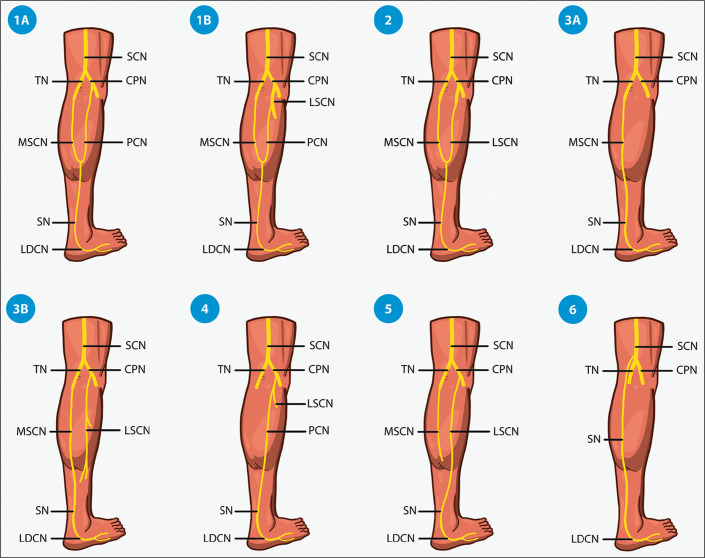
Classification of variations in the formation of the sural nerve. (SCN, sciatic nerve; TN, tibial nerve; CPN, common peroneal nerve; PCN, peroneal communicating nerve; SN, sural nerve; LDCN, lateral dorsal cutaneous nerve).

The proximal region of the sural nerve, composed of branches derived from the S1 and S2 (spinal) nerve roots, presents great anatomical and topographical variation^(7)^. According to Ramakrishnan et al.^(1)^, the formation of the sural nerve can be classified as one of six types (Figure 3), as outlined below.

• Type 1 (the most common type, seen in 51.5% of cases)
  - Type 1A: formed by the union of the MSCN, which originates from the tibial nerve, with the peroneal communicating nerve, which originates from the common peroneal nerve, in the proximal two thirds of the leg, accounting for 84.4% of all instances of type 1
  - Type 1B: formed by the union of the MSCN, which originates from the tibial nerve, with the communicating peroneal branch of the LSCN, which originates from the common peroneal nerve, in the proximal two thirds of the leg
• Type 2: formed by the union of the MSCN, which originates from the tibial nerve, with the LSCN, which originates from the common peroneal nerve
• Type 3
  - Type 3A: formed by the continuation of the MSCN in the absence of the peroneal communicating branch and the LSCN
  - Type 3B: formed by the continuation of the MSCN in the absence of the communicating peroneal branch, with or without the presence of the LSCN originating from the common peroneal nerve
• Type 4: formed by the peroneal communicating branch
• Type 5: formed by the LSCN alone, with the absence of or no contribution from the MSCN
• Type 6: formed by the nerve originating directly from the sciatic nerve.

The risk of iatrogenic injury is highest for types 3 and 4, whereas it is lowest for types 1 and 5^(3)^.

The systematic review conducted by Ramakrishnan et al.^(1)^ highlighted the variability in the site of union for sural nerve formation, which may occur anywhere between the popliteal fossa and the lateral malleolus. Their analysis, consistent with those of many previous studies, showed that the most prevalent site of union is in the lower half of the leg, with a pooled prevalence of 83.7% (95% CI: 0.765-0.899), whereas a proximal union in the upper half had a prevalence of only 16.3% (95% CI: 0.101-0.235). These findings do not align with those of Aktan Ikiz et al.^(8)^, who reported that in 60% of cases, the MSCN and LSCN united in the upper two thirds of the leg, whereas they united in the distal third in 10%. The lateral component was absent in 17% of cases, and the medial component was absent in 7%, with the two nerves following separate courses in another 7%^(8)^. Similarly, in an autopsy study of 76 individuals, Mahakkanukrauh et al.^(2)^ found that 67.1% of sural nerves were formed by the union of the MSCN and LSCN. Among those, the union occurred in the popliteal fossa in 5.9% of cases, in the middle third of the leg in 1.9%, in the lower third in 66.7%, and at or just below the ankle in 25.5%. In a minority of cases (0.7%), the sural nerve formed by the MSCN uniting with a different branch of the common peroneal nerve, whereas in 32.2% of specimens, it was a direct continuation of the MSCN. Aktan Ikiz et al.^(8)^ also measured the distance from the sural nerve to the lateral malleolus, reporting the mean distance to be 12.76 ± 8.79 mm from its most prominent part and 13.15 ± 6.88 mm from its tip. Those authors reported that the most common sensory distribution of the sural nerve was to the lateral side of the fifth toe (in 60.0% of cases), followed by the lateral two and a half toes (in 26.7%). Overall, whereas individual studies report anatomical variations, the systematic review conducted by Ramakrishnan et al.^(1)^ consolidated evidence indicating that the predominant pattern is a union in the lower half of the leg, emphasizing its anatomical variability and potential clinical implications for nerve localization and surgical approaches.

**Figure 4:**
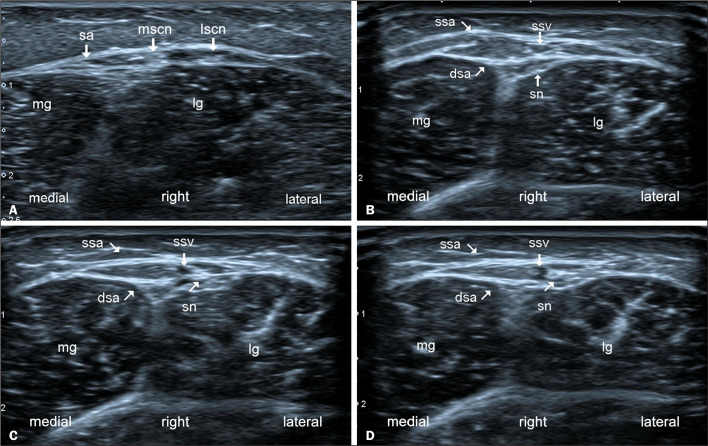
Axial ultrasonography depicting the anatomy of the sural nerve (sn) in the right calf, from proximal to distal (A-D). In A, the lscn and mscn are shown running superficial to the lateral and medial gastrocnemius muscles (lg and mg, respectively) and below the sural aponeurosis (sa). In B, the union of the two branches can be seen in a more distal position, from which they give rise to the sn and the deep sural aponeurosis (dsa). Note that the sa presents two layers: the dsa and the superficial sural aponeurosis (ssa). In C, the sn is shown crossing the dsa and subsequently running between the two aponeuroses. In D, the sn is shown crossing the dsa lateral to the small saphenous vein (ssv).

**Figure 5:**
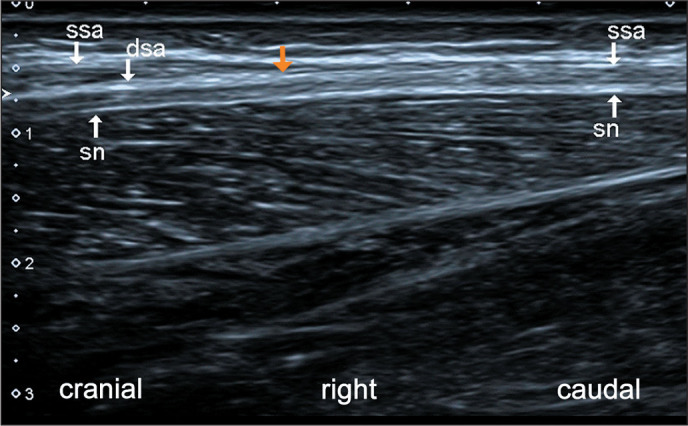
Longitudinal ultrasonography of the right calf, showing the sural nerve (sn) crossing the deep sural aponeurosis (dsa, orange arrow). Note that in the upper left part of the image there are two sural aponeuroses-the dsa and the superficial sural aponeurosis (ssa)-whereas there is only one (the ssa) in the upper right part.

The sural nerve typically enters the region of the small saphenous vein in the middle third of the calf, although the exact anatomy is highly variable.^(5)^ In that scenario, the nerve crosses the deep sural aponeurosis and lies between the superficial and deep aponeuroses, within the region of the small saphenous vein. That anatomical region is important because it is the site of entrapment of the sural nerve (Figures 4 and 5).

**Figure 6:**
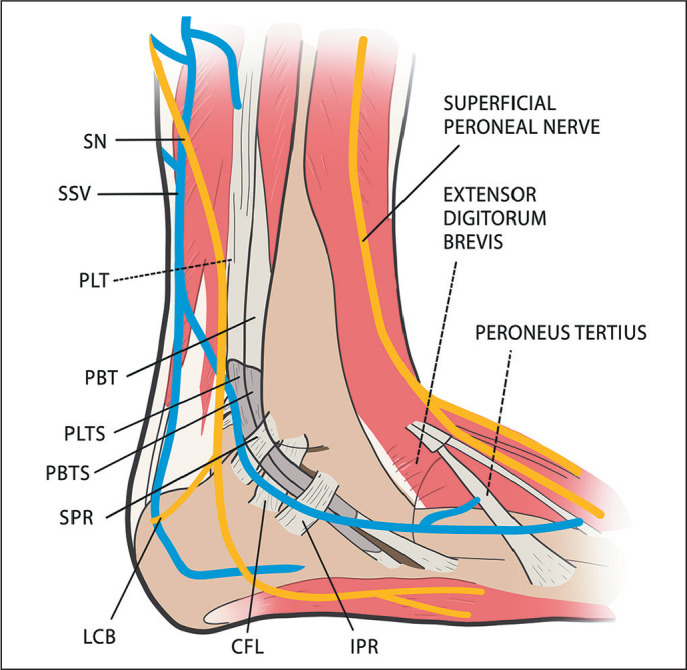
The anatomy of the lateral aspect of the ankle. (SN, sural nerve; SSV, small saphenous vein; PLT, peroneus longus tendon; PBT, peroneus brevis tendon; PLTS, peroneus longus tendon sheath; PBTS, peroneus brevis tendon sheath; SPR, superior peroneal retinaculum; LCB, lateral calcaneal branch (of the sural nerve); CFL, calcaneofibular ligament; IPR, inferior peroneal retinaculum).

In the distal third of the calf, after passing the sural aponeuroses, the sural nerve courses more superficially and laterally, adjacent to the small saphenous vein and lateral to the Achilles tendon (Figure 6). It should be borne in mind that the position of the sural nerve in relation to the small saphenous vein can vary, being anterior, posterior, or lateral to it. Identification of the exact course of the sural nerve facilitates surgical planning and helps avoid iatrogenic events^(5)^.

**Figure 7:**
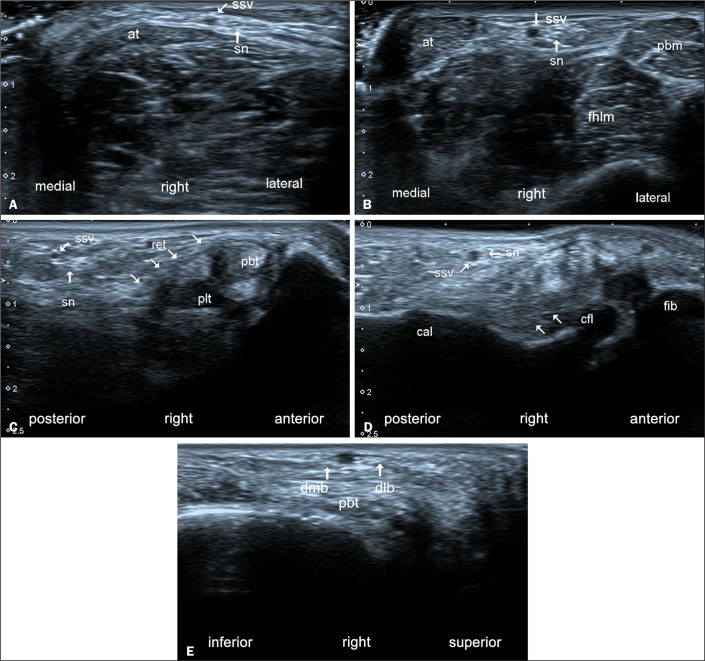
Ultrasonography in the axial plane (A,B) and in the coronal oblique plane (C-E), depicting the anatomy of the sural nerve (sn) in the right ankle and foot from proximal to distal (A-E). In A, the sn and the small saphenous vein (ssv) are seen lateral to the Achilles tendon (at). In B, the sn and ssv are located between the at and the flexor hallucis longus and peroneus brevis muscles (fhlm and pbm, respectively). More distally (C), the sn lies posterolateral to the peroneal retinaculum (ret) and superficial to the calcaneofibular ligament (cfl, in D). At the level of the base of the fifth metatarsal (E), just below the skin, the two branches of the lateral dorsal cutaneous nerve-the dorsomedial and dorsolateral branches (dmb and dlb, respectively)-run superficial to the peroneus brevis tendon (pbt). (cal, calcaneus; plt, peroneus longus tendon).

The sural nerve continues its course along the lateral aspect of the ankle, posterior to the peroneal tendons/retinaculum, and superficial to the calcaneofibular ligament. In that portion, it projects posteroinferiorly to the calcaneus (lateral calcaneal branch) and anterosuperiorly to the tibiotalar joint (lateral malleolus branch). The nerve then runs inferior to the lateral malleolus towards the base of the fifth metatarsal on the lateral surface of the foot; after the emergence of the lateral malleolar branch, it is referred to as the lateral dorsal cutaneous nerve, which, at the base of the fifth metatarsal, divides into two terminal branches-the medial dorsal branch and the lateral dorsal branch^(7)^-as depicted in Figures 6 and 7.

## ULTRASONOGRAPHY TECHNIQUE

Because of the small size and purely sensory nature of the sural nerve, ultrasonography seems more accurate than MRI for detecting sural nerve abnormalities in the ankle and foot^(7)^.

**Figure 8:**
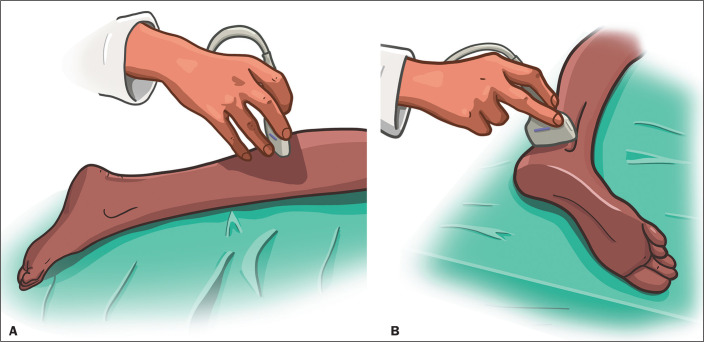
Proper positioning of the ultrasound transducer for examination of the sural nerve in the calf (A) and ankle (B).

To perform an ultrasound examination of the sural nerve, it is necessary to place the patient in the prone position with their knees extended and their feet hanging over the end of the examination table^(9)^. That positioning allows both sural nerves to be visualized from their origins to their distal ends^(9)^. A linear transducer, preferably a small one, is placed, with a large amount of gel, over the mid-lateral portion of the calf, which is a landmark for the small saphenous vein, the latter typically running lateral to the sural nerve^(9)^, as shown in Figure 8.

The ultrasound examination begins in the mid-calf with a short-axis approach, which allows the small saphenous vein and the adjacent sural nerve that runs within the subcutaneous tissues to be identified. The nerve and vein have variable positions in relation to each other. After the nerve has been located, the transducer is moved proximally and distally (the so-called elevator technique), following the nerve from the proximal portion of the calf to the ankle and foot, in order to rule out any anatomical variation. Care must be taken to maintain the transducer perpendicular to the axis of the nerve. Subtle changes in the size or internal architecture of the nerve are best evaluated by contralateral comparison. In the setting of pathologic states, abnormalities can be visualized on the long axis. Although the sural nerve does not show vascularity on a Doppler study, one is always performed in order to identify any local hypervascularity^(9)^.

Ricci et al.^(5)^ found that, during cross-sectional imaging of the calf, the contrast of the sural nerve in comparison with the surrounding tissues can be optimized by varying the angle of insonation while moving the transducer up and down the limb a short distance. According to the meta-analysis authored by Ramakrishnan et al.^(1)^, a typical sural nerve is 15 cm long and 0.3 cm in diameter. In another meta-analysis, Fisse et al.^(10)^ showed that the cross-sectional area (CSA) of the sural nerve is 2.4 mm^2^ at the level of the heads of the gastrocnemius muscle. The sural nerve is formed by the medial cutaneous branch of the tibial nerve and the lateral cutaneous branch of the common fibular nerve, with this junction occurring at varying levels of the calf. Measurements taken above or below the formation of the sural nerve can result in slight differences in CSA. In the Fisse et al.^(10)^ review, the univariate and multivariate regression analyses revealed no significant associations with age, height, or weight. However, the authors found that CSA values were higher in studies conducted in New Zealand than in those conducted in Europe, highlighting notable regional differences. The low heterogeneity in sural nerve CSA may be attributable to interindividual anatomical variations.

## SURGICAL PLANNING

The sural nerve is the most common donor nerve used for reconstruction because it is relatively long and easy to remove. However, it is put at risk by the incisions commonly used in lateral reconstructions, calcaneal/peroneal tendon repair or tenorrhaphy, subtalar arthrodesis, and the fixation of distal fibular fractures^(8)^. Reportedly, 7.5% of surgical procedures for the treatment of varicose veins in the United Kingdom result in sural nerve damage; endovascular vein ablation procedures carry a risk of sural nerve injury, especially laser ablation, for which the risk of such injury is reported to be 2%^(2^,^5)^. Among surgical procedures performed for the repair of Achilles tendon injuries, 13% result in injury to the sural nerve^(5)^. Sutures placed near the proximal lateral border of the Achilles tendon can damage the sural nerve^(10)^. Because of its proximity to the small saphenous vein, the sural nerve is also at risk of injury during the following procedures^(5)^: dissection of the saphenopopliteal junction; stripping of the small saphenous vein; phlebectomy of the small saphenous vein and its tributaries; and thermal ablation of the small saphenous vein. Knowledge of the anatomy of the sural nerve minimizes such surgical complications^(8)^. According to Rama-krishnan et al.^(1)^, it is recommended that the anatomy of the sural nerve be evaluated by ultrasonography prior to nerve conduction tests. Despite various reports that the sural nerve is asymmetrical, there is still some disagreement in the literature. Mahakkanukrauh et al.^(2)^ detected bilateral asymmetry in the pattern of formation of the sural nerve in 80.4% of individuals evaluated postmortem. However, Ramakrishnan et al.^(1)^ found that the prevalence of symmetric formation of the sural nerve across studies in the literature was 64.1%^(1)^. Nevertheless, asymmetry in the formation of the sural nerve is not uncommon and requires attention from a medical team.

### Limitations

Like all diagnostic imaging methods, ultrasonography has some limitations. It can be of limited utility in certain scenarios, such as in obese patients, as well as in those with hematoma or edema of the subcutaneous tissue; in such patients, it is difficult to visualize the sural nerve on ultrasonography, especially if the examination is performed by an inexperienced operator^(9)^.

## CONCLUSION

The sural nerve, due to its superficial nature and complex anatomical variations, plays a significant role in both diagnostic and therapeutic procedures. Ultrasonography has proven to be an invaluable tool in visualizing this nerve, aiding in precise surgical planning and minimizing the risk of iatrogenic injury. Understanding the intricate anatomy of the sural nerve, including its variations and its relationship with surrounding structures like the small saphenous vein, is crucial for clinicians to perform effective and safe interventions. However, while ultrasonography offers many advantages, such as being noninvasive and cost-effective, its utility can be limited in certain patient populations, underscoring the importance of operator expertise and the potential need for supplementary imaging methods in challenging cases. Ultimately, detailed knowledge of sural nerve anatomy and careful preoperative planning can significantly improve patient outcomes after surgical procedures involving one or both of the lower limbs.
